# Radiotherapy for Large Mucosa-Associated Lymphoid Tissue Lymphoma of the Rectum: A Case Report

**DOI:** 10.7759/cureus.80588

**Published:** 2025-03-14

**Authors:** Yukihiko Yoshimatsu, Mai Anakura, Munenori Ide, Takuma Ishizaki, Hiroki Kiyohara

**Affiliations:** 1 Department of Radiation Oncology, Japanese Red Cross Maebashi Hospital, Maebashi, JPN; 2 Department of Radiation Oncology, Gunma University Graduate School of Medicine, Maebashi, JPN; 3 Department of Pathology, Maebashi Red Cross Hospital, Maebashi, JPN; 4 Department of Hematology, Japanese Red Cross Maebashi Hospital, Maebashi, JPN

**Keywords:** bloody stools, conventional radiotherapy, gastrointestinal, large rectal mass, large-sized lymphoma, malt, radiation therapy, rectal mucosa-associated lymphoid tissue lymphoma, volumetric modulated arc therapy (vmat)

## Abstract

Mucosa-associated lymphoid tissue (MALT) lymphomas may arise at various sites, but MALT lymphoma of the rectum is rare. Several treatment options are available for MALT lymphoma of the rectum; however, no standard treatment has been clearly defined. Herein, we report a case of large-sized MALT lymphoma of the rectum that was successfully treated with radiotherapy. The patient presented to the hospital with a complaint of bloody stools and, after a biopsy, was diagnosed with MALT lymphoma of the rectum. Before treatment, the tumor size was 50 × 62 × 70 mm. Radiotherapy was administered at a total dose of 39.6 Gy in 22 fractions, and the patient achieved a complete metabolic response two months after treatment. The patient was followed up for 29 months after radiotherapy, and no recurrence or severe adverse events were observed. This report demonstrates that radiotherapy may be a definitive treatment option for large-sized MALT lymphomas of the rectum.

## Introduction

According to the 2022 World Health Organization (WHO) classification, mucosa-associated lymphoid tissue (MALT) lymphoma is classified as a marginal zone lymphoma (MZL) [1]. It arises from MALT, such as the gastrointestinal tract (especially the stomach), ocular appendages, lungs, salivary glands, thyroid, and breast, with the highest frequency in the stomach [2]. Primary lymphomas of the colorectum are rare and account for only 0.2% of all colorectal malignancies [3]. MALT lymphomas of the colorectum account for 1.6% of all MALT lymphomas [4].

Treatment options for primary MALT lymphomas of the gastrointestinal tract excluding the stomach include *Helicobacter pylori* eradication therapy, surgery, endoscopic mucosal resection (EMR), chemotherapy, radiotherapy, and observation [5]. However, a standard treatment has not been established, and the optimal prescribed doses for radiotherapy have not been clearly defined. Here, we report a case of large-sized MALT lymphoma of the rectum that was successfully treated with radiotherapy.

## Case presentation

**Figure 1:**
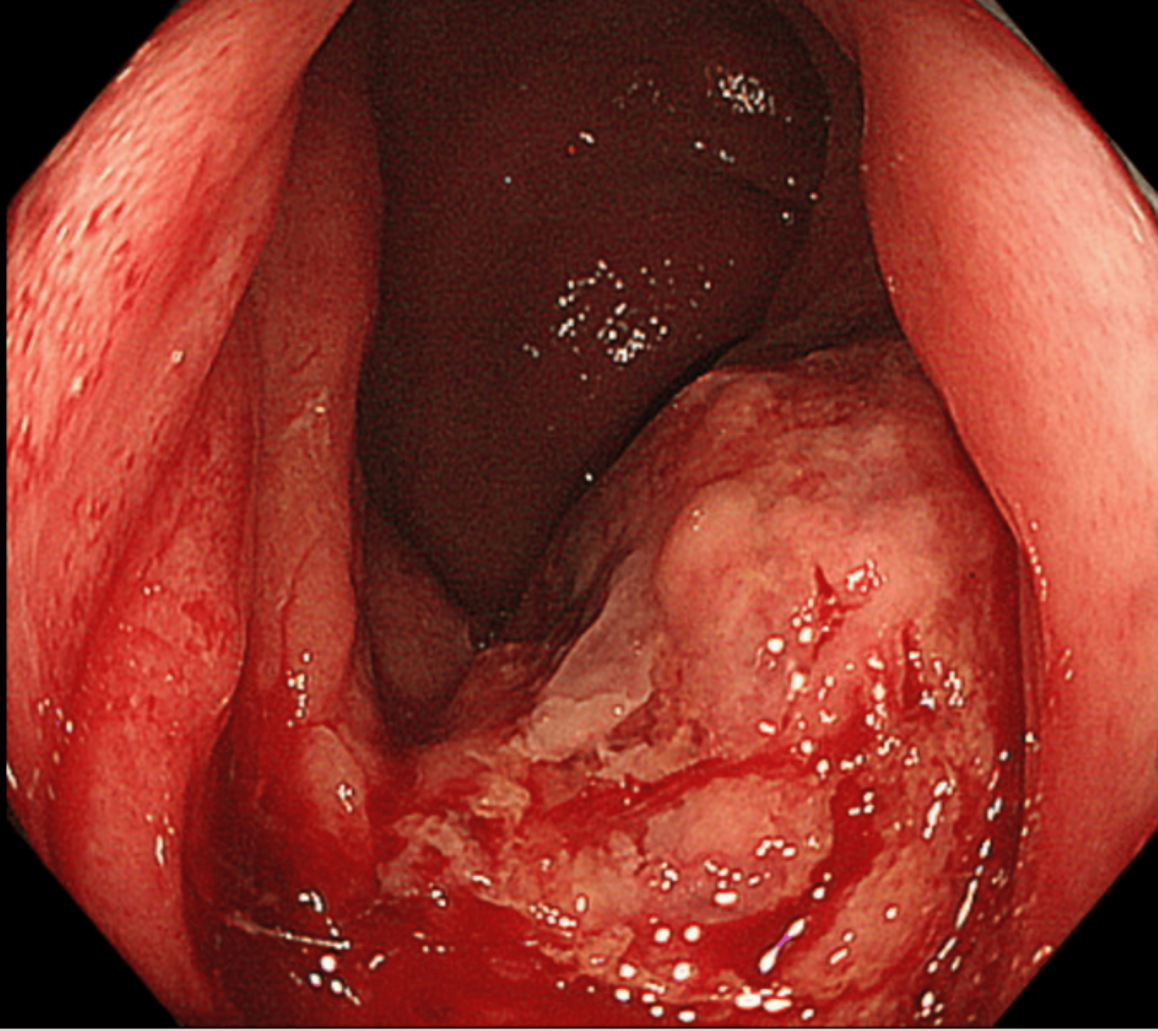
Colonoscopy image before radiotherapy showing an elevated tumor on the posterior wall of the lower rectum, 1 cm from the anal verge.

**Figure 2:**
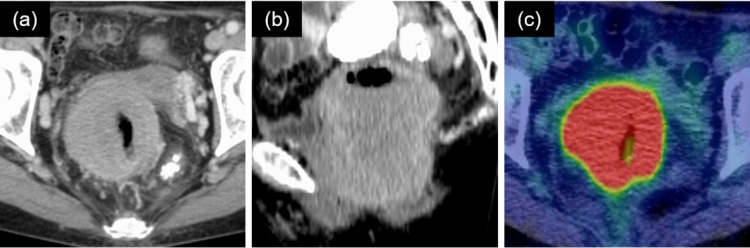
Images before radiotherapy. (a) Axial view of CT. (b) Sagittal view of CT. (c) Axial view of FDG-PET/CT. SUVmax: 20.5. CT: computed tomography; FDG-PET/CT: 18F-fluorodeoxyglucose-positron emission tomography/CT; SUVmax: maximum standardized uptake value

**Figure 3:**
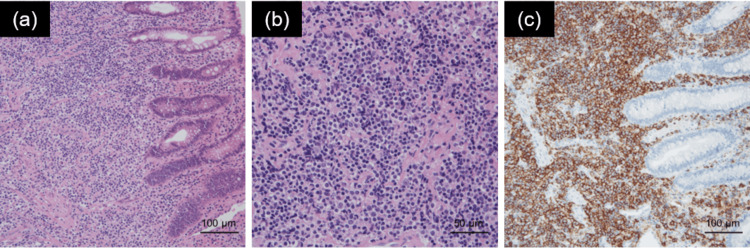
Pathological findings in the biopsy specimen. (a, b) Proliferation of medium- to large-sized bright cytoplasmic lymphocytes on hematoxylin-eosin staining. (c) CD20-positive specimen. Original magnification: ×200 (a), ×400 (b), and ×200 (c).

The patient was a 76-year-old woman who was aware of bloody stools that she had left untreated for one year. At that time, the patient was not aware of B-symptoms. She then went to a local hospital and underwent lower gastrointestinal endoscopy, which revealed a friable 4 cm protruding submucosal tumor on the posterior wall of the lower rectum, 1 cm from the anal verge (Figure 1). The patient was referred to our institution with the suspicion of a malignant tumor. Contrast-enhanced computed tomography (CT) revealed circumferential wall thickening from the lower rectum to the anal canal, and the tumor size was 50 × 62 × 70 mm (Figure 2). Lower gastrointestinal endoscopic and transanal tumor biopsies were performed. Histopathological examination revealed the proliferation of medium- to large-sized bright cytoplasmic lymphocytes, CD20 positivity, no obvious lymphoepithelial lesions, poor monoclonality of kappa and lambda light chain expression, and a low Ki-67 labeling index of 20% (Figure 3). On 18F-fluorodeoxyglucose (FDG)-positron emission tomography (PET)/CT, FDG accumulation was observed with a maximum standardized uptake value (SUVmax) of 20.5 in the tumor on the rectum (Figure 2) and no obvious abnormal accumulation in other parts of the body. Based on these findings, the patient was diagnosed with stage I localized primary MALT lymphoma of the rectum (Lugano classification).

**Figure 4:**
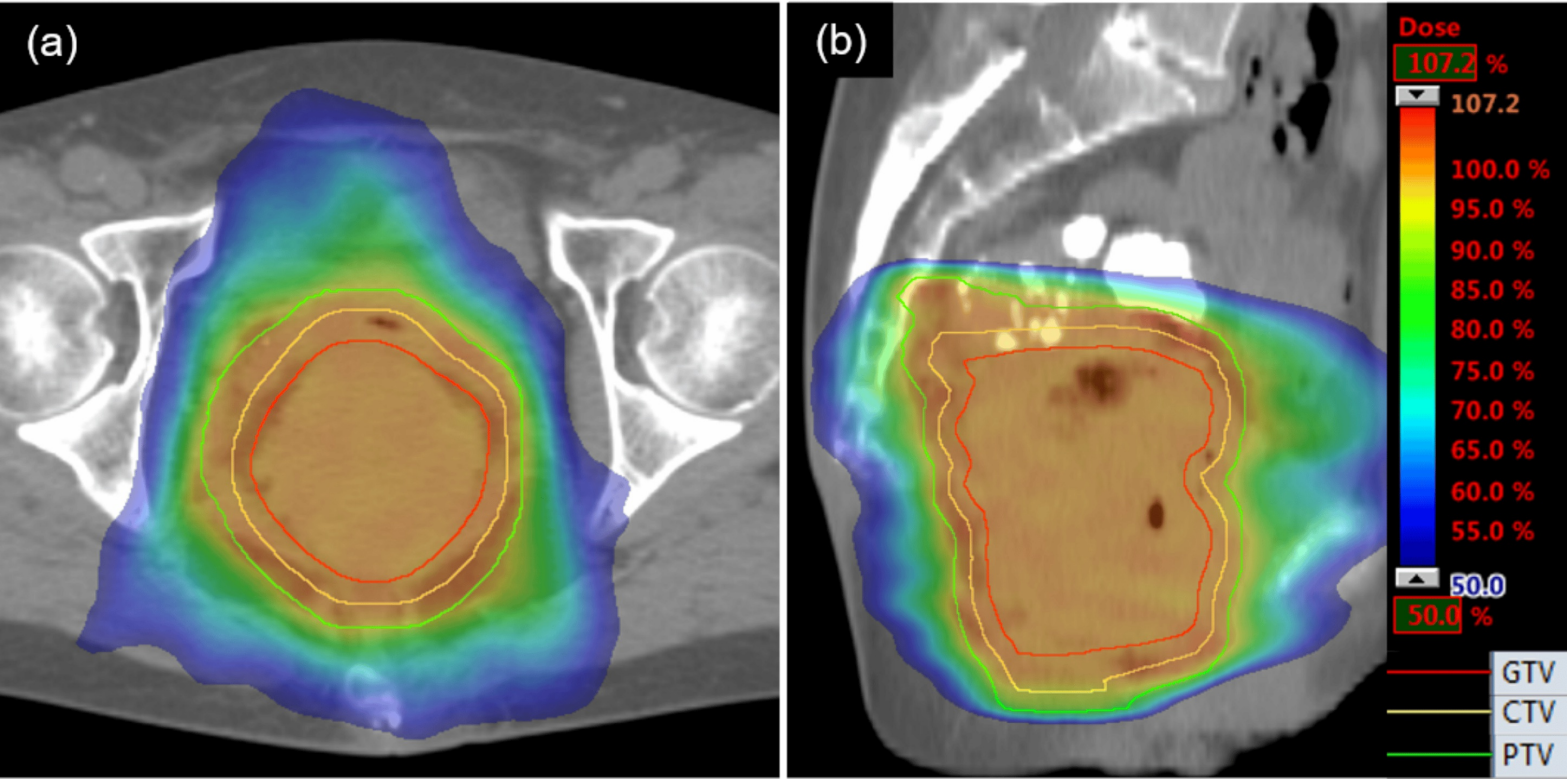
Images of dose distribution. (a) Axial view of CT. (b) Sagittal view of CT. CT: computed tomography; GTV: gross tumor volume; CTV: clinical target volume; PTV: planning target volume

Therefore, we decided to treat the patient with radiotherapy. The gross tumor volume (GTV) was delineated using enhanced CT images taken consecutively with the treatment-planning CT. Clinical target volume (CTV) was delineated to the entire rectal mucosal area, including the GTV. The planning target volume (PTV) was delineated by adding a 5-mm margin to the CTV. We used Varian's Clinac iX for treatment. Irradiation was conducted using volumetric modulated arc therapy (VMAT) with a total dose of 39.6 Gy delivered in 22 fractions five times a week (Figure 4). During each irradiation session for image-guided radiation therapy, the spatial reproducibility of the three-dimensional position of the center of irradiation at the time of treatment planning and at the time of irradiation had been confirmed to be within 5 mm by cone-beam CT images in the treatment room. An additional treatment plan CT was performed at the 14th irradiation session, and the tumor size was reduced to 38 × 41 × 63 mm. Consequently, the patient received treatment with a revised plan in the last six sessions.

**Figure 5:**
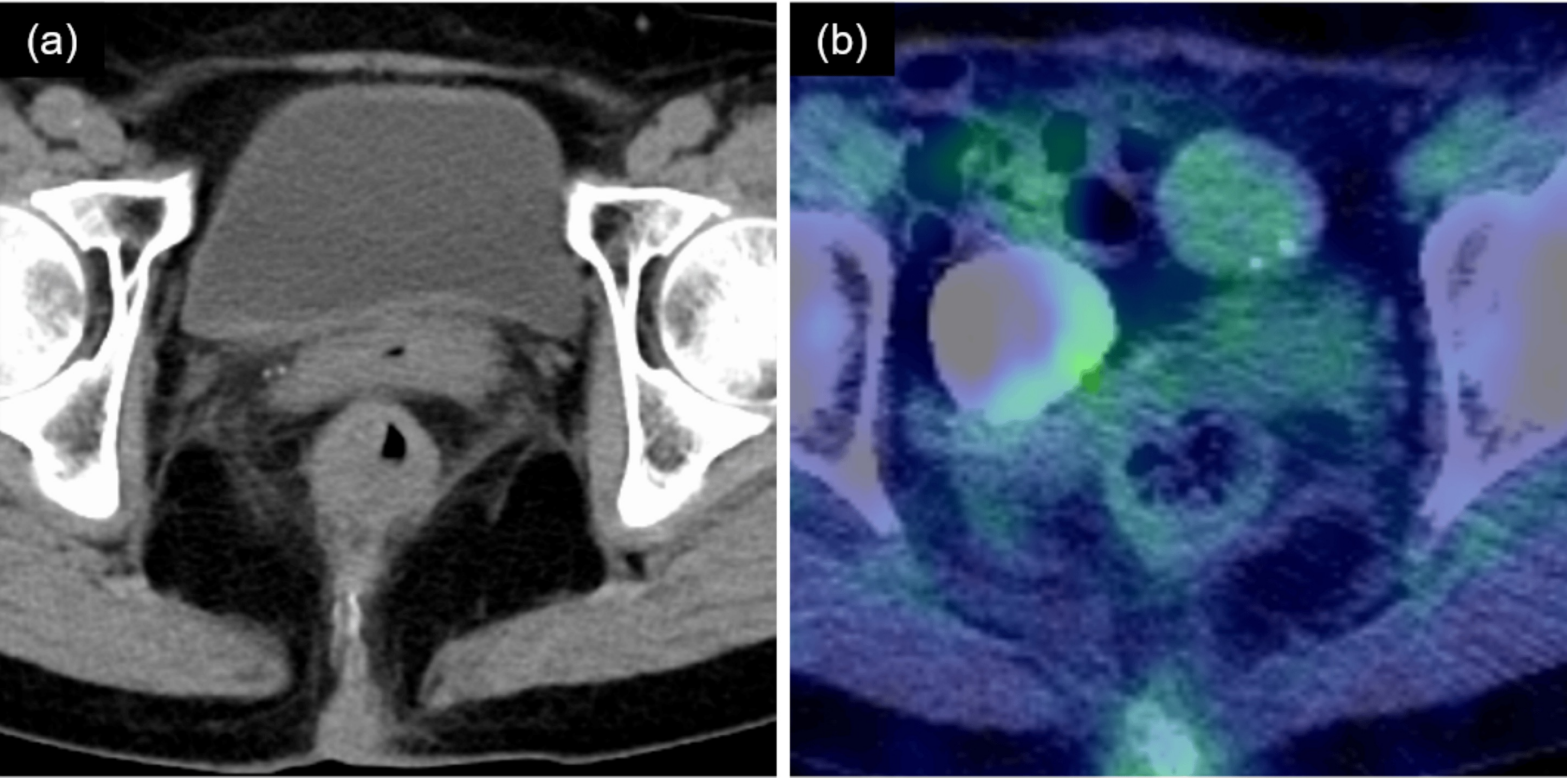
Images after radiotherapy. (a) Axial view of CT. (b) Axial view of FDG-PET/CT. SUVmax: 2.5. CT: computed tomography; FDG-PET/CT: 18F-fluorodeoxyglucose-positron emission tomography/CT; SUVmax: maximum standardized uptake value

The bloody stools stopped at 25.2 Gy and did not recur. Adverse events were evaluated using the Common Terminology Criteria for Adverse Events (CTCAE) version 5.0. At the end of the treatment, the patient had grade 1 dermatitis, which improved three weeks after irradiation. No other acute adverse events were observed. There were no abnormalities in defecation after treatment. FDG-PET/CT at two months after treatment showed a decrease in SUVmax to 2.5, diagnosed as a complete metabolic response. CT at 20 months after treatment showed no apparent relapse (Figure 5). At the 29-month follow-up after irradiation, the patient was alive with no adverse events.

## Discussion

Several treatment options are available for MALT lymphoma of the rectum, including *Helicobacter pylori* eradication therapy, surgery, EMR, chemotherapy, and observation. Radiotherapy is one such therapy, and its effectiveness has been reported in several studies (Table 1) [6-17]. The optimal dose has not been established; however, in these reports, radiotherapy was administered at 30-40 Gy, and in many cases, complete remission was achieved.

**Table 1:** Reported cases of MALT lymphoma of the rectum that underwent radiotherapy. MALT: mucosa-associated lymphoid tissue; NA: not applicable; CR: complete remission; ※: including one case of duodenal primary

Year	First author (reference)	Age, years	n	Size, mm	Total dose, Gy	Fraction	Outcome	Follow-up, months	Late toxicities
2001	Tsang [6]	NA	1	NA	35	NA	CR	>48	None
2008	Yamashita [7]	NA	1	NA	30	20	CR	NA	NA
2008	Kobayashi [8]	26	1	100	40	NA	CR	24	NA
2008	Foo [9]	60	1	NA	45	25	CR	41	None
2012	Okamura [10]	56-65	3	10-20	30	20	CR	13-75	NA
2012	Akasaka [11]	57	1	>30	30	NA	CR	9	NA
2017	Hatayama [12]	28	1	NA	30.6	17	CR	9	None
2017	Hayakawa [13]	74	1	≤3	30	15	CR	60	NA
2020	Watanabe [14]	38-89	4^※^	NA	30	15-20	CR	>60	None
2022	Katano [15]	50-67	3	10-30	30	15	CR	56-59	None
2023	Zhang [16]	29	1	NA	30	15	CR	3	NA
2025	Present case	76	1	70	39.6	22	CR	29	None

MALT lymphoma of the rectum is often observed in small sizes [18], and surgery or EMR is indicated in such cases [5]. Reports of large tumors, such as those in the present case, are rare. Kobayashi et al. reported a patient treated with radiotherapy for MALT lymphoma of the rectum with a tumor size of 100 mm that achieved complete remission after a total dose of 40 Gy [8]. Furthermore, Isobe et al. stated that in their multicenter phase II study for stage IE MALT lymphoma not originating in the stomach, they adopted 36 Gy in 20 fractions for tumors <6 cm and 39.6 Gy in 22 fractions for those ≥6 cm in size; local control and progression-free survival rates were 97.3% and 91.9% at three years, respectively [19]. In addition, the tumor was large and located 1 cm from the anal verge in the present case. If surgery had been performed, surgery would have required a total rectal resection with colostomy as a curative treatment, and the quality of life in this patient would have declined. From the above, we selected radiotherapy owing to the tumor size of >6 cm, with a total dose of 39.6 Gy in 22 fractions, for this patient.

No studies have described the need for changes in treatment plans according to tumor shrinkage during the treatment period. In the present case, we modified our treatment plan once the tumor shrank and changed the plan at the 17th treatment session. We believe that re-planning should be considered for large tumors, as seen in the present case.

MALT lymphoma is classified as an MZL. Histological transformation to large B-cell lymphomas is reported in MZL. The five- and 10-year cumulative incidence of histological transformation was 2.7% and 3.6% [20]. In the present case, the patient had not relapsed at the 29-month follow-up after irradiation. The patient should be followed up with attention to the possibility of histological transformation occurring at recurrence in the future.

## Conclusions

There are no clearly defined treatment strategies for MALT lymphomas of the rectum. We treated the patient with radiotherapy as a definitive treatment for large-sized MALT lymphoma of the rectum. The tumor was well controlled, with no severe adverse events during the 29-month follow-up period. This suggests that radiotherapy is an effective treatment option for large-sized MALT lymphomas of the rectum.

## References

[1] Alaggio R, Amador C, Anagnostopoulos I (2022). The 5th edition of the World Health Organization classification of haematolymphoid tumours: lymphoid neoplasms. Leukemia.

[2] Olszewski AJ, Castillo JJ (2013). Survival of patients with marginal zone lymphoma: analysis of the Surveillance, Epidemiology, and End Results database. Cancer.

[3] Dionigi G, Annoni M, Rovera F (2007). Primary colorectal lymphomas: review of the literature. Surg Oncol.

[4] Hahn JS, Kim YS, Lee YC, Yang WI, Lee SY, Suh CO (2003). Eleven-year experience of low grade lymphoma in Korea (based on REAL classification). Yonsei Med J.

[5] Won JH, Kim SM, Kim JW, Park JH, Kim JY (2019). Clinical features, treatment and outcomes of colorectal mucosa-associated lymphoid tissue (MALT) lymphoma: literature reviews published in English between 1993 and 2017. Cancer Manag Res.

[6] Tsang RW, Gospodarowicz MK, Pintilie M, Bezjak A, Wells W, Hodgson DC, Crump M (2001). Stage I and II MALT lymphoma: results of treatment with radiotherapy. Int J Radiat Oncol Biol Phys.

[7] Yamashita H, Nakagawa K, Asari T, Murakami N, Igaki H, Ohtomo K (2008). Radiotherapy for 41 patients with stages I and II MALT lymphoma: a retrospective study. Radiother Oncol.

[8] Kobayashi T, Takahashi N, Hagiwara Y (2008). Successful radiotherapy in a patient with primary rectal mucosa-associated lymphoid tissue lymphoma without the API2-MALT1 fusion gene: a case report and review of the literature. Leuk Res.

[9] Foo M, Chao MW, Gibbs P, Guiney M, Jacobs R (2008). Successful treatment of mucosa-associated lymphoid tissue lymphoma of the rectum with radiation therapy: report of a case. Dis Colon Rectum.

[10] Okamura T, Suga T, Iwaya Y (2012). Helicobacter pylori-negative primary rectal MALT lymphoma: complete remission after radiotherapy. Case Rep Gastroenterol.

[11] Akasaka R, Chiba T, Dutta AK (2012). Colonic mucosa-associated lymphoid tissue lymphoma. Case Rep Gastroenterol.

[12] Hatayama Y, Aoki M, Kawaguchi H (2017). Safe and successful birth following pelvic radiotherapy for rectal mucosa-associated lymphoid tissue lymphoma: a case report. J Med Case Rep.

[13] Hayakawa T, Nonaka T, Mizoguchi N (2017). Radiotherapy for mucosa-associated lymphoid tissue (MALT) lymphoma of the rectum: a case report. Clin J Gastroenterol.

[14] Watanabe S, Ogino I, Hata M (2020). Radiotherapy for non-gastric intestinal versus gastric MALT lymphoma: a comparison of treatment outcomes. Blood Res.

[15] Katano A, Takeuchi K, Yamashita H (2022). Radiotherapeutic outcomes for localized primary rectal mucosa-associated lymphoid tissue lymphoma: a consecutive case series of three patients. Cureus.

[16] Zhang JY, Fu BZ, Yin ZK, Li J (2023). Primary rectal mucosa-associated lymphoid tissue lymphoma masquerading as proctitis. Rev Esp Enferm Dig.

[17] Alvi AT, Shankar M (2023). A rare case of primary extra-nodal marginal zone lymphoma of mucosa-associated lymphoid tissue (MALT) in the rectum. Cureus.

[18] Ishikawa E, Nakamura M, Satou A, Shimada K, Nakamura S (2022). Mucosa-associated lymphoid tissue (MALT) lymphoma in the gastrointestinal tract in the modern era. Cancers (Basel).

[19] Isobe K, Kagami Y, Higuchi K (2007). A multicenter phase II study of local radiation therapy for stage IEA mucosa-associated lymphoid tissue lymphomas: a preliminary report from the Japan Radiation Oncology Group (JAROG). Int J Radiat Oncol Biol Phys.

[20] Bommier C, Link BK, Gysbers BJ (2024). Transformation in marginal zone lymphoma: results from a prospective cohort and a meta-analysis of the literature. Blood Adv.

